# Long-term survival of hybrid total hip replacement for prior failed proximal femoral nail antirotation: a retrospective study with a median 10-year follow-up

**DOI:** 10.1186/s12891-022-05827-3

**Published:** 2022-09-16

**Authors:** Lin Wang, Minji Yu, Yaodong Zhang, Shuxin Wang, Mingdong Zhao, Mingliang Yu, Si Li, Songtao Gao, Min Xiong, Weiguang Yu

**Affiliations:** 1 Department of Orthopedics, Henan Provincial People’s Hospital; Zhengzhou University People’s Hospital, No.7, Weiwu Road, Jinshui District, Zhengzhou, Henan 450003 China; 2 Department of Traditional Chinese Medicine, The First Affiliated Hospital, Sun Yat-Sen University, No. 58, Zhongshan 2nd Road, Yuexiu District, Guangzhou, 510080 China; 3 Department of Anesthesiology, The First Affiliated Hospital, Sun Yat-Sen University, No. 58, Zhongshan 2nd Road, Yuexiu District, Guangzhou, 510080 China; 4 Department of Orthopedics, The First Affiliated Hospital, Sun Yat-Sen University, No. 58, Zhongshan 2nd Road, Yuexiu District, Guangzhou, 510080 China; 5 Department of Orthopaedics, Jinshan Hospital, Fudan University, Longhang Road No. 1508, Jinshan District, Shanghai, 201508 China; 6 Department of Anesthesiology, Wuhan Fourth Hospital; Puai Hospital, Tongji Medical College, Huazhong University of Science and Technology, No.473, Hanzheng Street, Qiaokou District, Wuhan, 430033 China

**Keywords:** Failure, Total hip replacement, Revision, Conversion, Fracture

## Abstract

**Background:**

Hybrid total hip replacement (THR) is commonly used in the management of proximal femur fractures in elderly individuals. However, in the context of the revision, the literature on hybrid THR is limited, and differences in the long-term survival outcomes reported in the literature are obvious. This retrospective study aimed to evaluate the long-term survival of hybrid THR for failed proximal femoral nail antirotation (PFNA) in elderly individuals aged ≥ 75 years.

**Methods:**

An observational cohort of 227 consecutive individuals aged ≥ 75 years who experienced hybrid THRs following prior primary PFNAs was retrospectively identified from the Joint Surgery Centre, the First Affiliated Hospital, Sun Yat-sen University. Implant survival was estimated using the Kaplan–Meier method. The primary end point was the implant survivorship calculated using the Kaplan–Meier method with revision for any reason as the end point; secondary end points were the function score measured using the modified Harris Hip Score (mHHS) and the incidence of main orthopaedic complications.

**Results:**

In total, 118 individuals (118 THRs) were assessed as available. The median follow-up was 10 (3–11) years. The 10-year survivorship with revision for any reason as the endpoint was 0.914 (95% confidence interval [CI], 0.843–0.960). The most common indication for revision was aseptic loosening (70.0%), followed by periprosthetic fracture (30.0%). At the final follow-up, the median functional score was 83.6 (79.0–94.0). Among the 118 patients included in this study, 16 experienced 26 implant-related complications. The overall incidence of key orthopaedic complications was 13.5% (16/118).

**Conclusion:**

For patients aged ≥ 75 years old with prior failed PFNAs, hybrid THR may yield satisfactory long-term survival, with good functional outcomes and a low rate of key orthopaedic complications.

## Background

The challenge of dealing with failed proximal femoral nail antirotation (PFNA) is a well-established climb and continues [1–3]. As longevity has increased in China, the incidence of failed PFNA is bound to increase, especially in individuals characterised by advancing age, osteoporosis, and multiple comorbidities, which already poses a great threat to the patient's quality of life, despite the lack of Chinese-specific data [2, 3]. Accordingly, revision procedures for prior failed PFNA may present an increasing trend [1]. Recently, several studies [1–3] have shown a growing rate of revisions for failed PFNA. Disappointingly, however, options including hybrid, cemented, and uncemented total hip replacements (THRs) used to revise a failed PFNA remain controversial [3–5]. Furthermore, different THRs used in this context tend to result in specific complications [6]. Advocates of hybrid THR perceive benefits regarding function score and orthopaedic complications when compared to cemented or uncemented THR [7–9]. Hybrid THR increases the early stability of the femoral component after revision surgery and has frequently been used in an attempt to increase early weight bearing, with apparently encouraging results [7, 10]. Nevertheless, the cemented femoral component of hybrid THR may have a great destructive effect on the surrounding bone tissue [11]. Furthermore, there are growing concerns that cemented femoral components have greater associated cement-related complications than uncemented femoral components and could increase the risk of cement-induced osteolysis, which frequently results in prosthesis failure [7, 12]. Although failed PFNA converted to THR has been a recognised treatment strategy, the inappropriate decision as to which type of THR (hybrid, cemented, and uncemented) is the optimum treatment in elderly individuals may lead to a marked difference in treatment outcomes [13, 14].

Currently, there is an ongoing debate on the utilisation of hybrid vs. uncemented THRs [7, 15, 16]. With the development of cement fixation techniques, there has been a growing use of hybrid THR in China, particularly in individuals aged ≥ 75 years. However, concerns related to the long-term implant survival of hybrid THRs remain [1, 3]. Furthermore, there remain no definite long-term follow-up results for patients in China experiencing a hybrid THR following prior PFNA failures that are sufficient to show the superiority of hybrid THR, especially for individuals aged ≥ 75 years. Hence, we executed this retrospective review to assess the long-term outcomes of individuals aged ≥ 75 years old with prior PFNA failures who underwent a hybrid THR.

## Methods

### Study population

From August 2010 to December 2019, consecutive elderly individuals aged ≥ 75 years old experiencing a hybrid THR following prior failed PFNA were retrospectively reviewed from three joint trauma centres. The median volume of THR revisions per year at each centre was 20 procedures (range, 11–32). The product details of PFNA and hybrid THR are presented in Table 1. The type of and reason for revision PFNA were identified based on electronic medical records. The comorbidities of the included patients were assessed using the Charlson comorbidity index (CCI). Key inclusion criteria consisted of elderly individuals aged ≥ 75 years and individuals experiencing initial PFNA fixation, followed by hybrid THR revision. Key exclusion criteria were as follows: lacking demographic data (i.e., diagnosis, type of fixation, implant details); lack of follow-up data; hip deformity; loss of independent athletic ability; previous contralateral intertrochanteric fractures; disorders of the nervous system of the lower extremities; advanced tumours; active infectious diseases (i.e., sepsis, interstitial pneumonia, osteomyelitis, and meningitis); mental abnormalities (i.e., schizophrenia, mental retardation, severe depression, attention deficit hyperactivity disorder); long-term dialysis or drug therapy (i.e., renal failure, immunosuppressants, antithyroid drugs); and failure to abide by the follow-up plan.

**Table 1:** Product details of PFNA and hybrid THR

	Stem^a^	Cup^a^	PFNA
Hybrid THR(*n* = 118)	cemented stem with ceramic femoral head	uncemented monoblock trabecular metal cup^b^	Synthes, Solothurn, Switzerland

*PFNA* Proximal femoral nail antirotation, *THR* Total hip replacement

^a^Zimmer, Warsaw, Indiana

^b^made from highly porous tantalum with a polyethylene liner

### Surgical procedures

We used the previous surgical incision to remove the PFNA device. After the removal of the PFNA device, we routinely measured the length between the lesser trochanter and the distal tip of the main nail after the removal of the PFNA device. The length of the stem was greater than or equal to the length of the main nail measured previously, avoiding the phenomenon of periprosthetic fracture associated with local stress concentration of the stem. All conversion procedures were executed through a lateral approach. A uncemented cup was inserted in accordance with manufacturer’s instructions. We performed two truncations of the femoral neck at the junction of the femoral head and neck and at the base of the femoral neck, removed the glenoid labrum, and polished the acetabulum until the bone surface oozed blood. With a valgus angle of 40° to 45° and an anteversion angle of 15°, the acetabulum cup was punched into the acetabulum, and the liner was installed.

Cemented stems were inserted using third-generation cementing techniques. During stem insertion, subperiosteal osteolysis, resorption of the femoral calcar, proximal femoral bone defect, and disruption of the integrity of the greater trochanter are of concern. There are the following technical points: long-stem prosthesis is selected; wedge-shaped bone masses of the femoral neck can be used to reconstruct the integrity of the greater trochanter; the cancellous bone of the femoral head and the cortical bone of the femoral neck are made into 2–3 mm bone fragments for bone grafting; cortical defects can be bound with steel cables and covered with metal mesh; the proximal femoral medullary cavity should be thoroughly cleaned; long guide needle is located in the center of the medullary cavity in the distal femur and near the lateral cortical bone in the proximal femur; the prepared 2–3 mm bone fragments were implanted into the proximal femoral medullary cavity and the residual lateral screw holes after PFNA removal (due to the coverage of periosteum or musculoaponeurotic layer, the medial screw holes rarely had cement leakage).

### Outcomes and variables

The primary end point was the implant survivorship calculated using the Kaplan–Meier method with revision for any reason as the endpoint. Revision was defined as exchange or removal of full or partial implants including the acetabulum, cup and liner for any reason [17], irrespective of component adjustment. Each revision was symptomatic. Indications for conversion to hybrid THR involved instability, mechanical failure, and both. Instability was defined as screw loosening, unacceptable displacement of the fracture site, nonunion, tendency of dislocation. The secondary endpoints were the function score measured using the modified Harris Hip Score (mHHS: range, 0–100, with higher scores indicating better function) and the incidence of main orthopaedic complications (aseptic loosening, dislocation, and periprosthetic fracture). mHHS = 70 was regarded as the threshold for failure [18]. The cemented stem was classified as definite loosening if radiolucent lines with distinct migration measured on two consecutive radiographs were present [19]. Loosening of the acetabular component was defined as a continuous radiolucent line greater than 2 mm in width on both the anteroposterior and the lateral radiographs compared with the immediate postoperative images [20]. Image data collected at every follow-up were reviewed centrally. At the end of the study, the secondary endpoints were confirmed by the two coauthors (WY and MX). Main orthopaedic complications and deaths at any time during the follow-up period were recorded. Patients underwent evaluation on the day following revision PFNA and continued until the occurrence of revision THR, death, or study deadline, whichever came first. Patients were followed up at an interval of 2–3 months, either in person or by telephone.

### Statistical analysis

The survival for hybrid THRs used in this study was calculated using the Kaplan–Meier survival with 95% confidence interval (CI). The primary endpoint analysis using death as a competing risk was conducted with revision THR for any reason as an endpoint. Considering that the functional outcome data calculated using the mHHS were normally distributed during follow-up, the preoperative and postoperative mHHS were compared using a paired Student’s t test. The alpha level was set at 0.05 when comparing differences between function scores. Data analyses were performed using SAS 9.4 (SAS Institute).

## Results

**Fig. 1:**
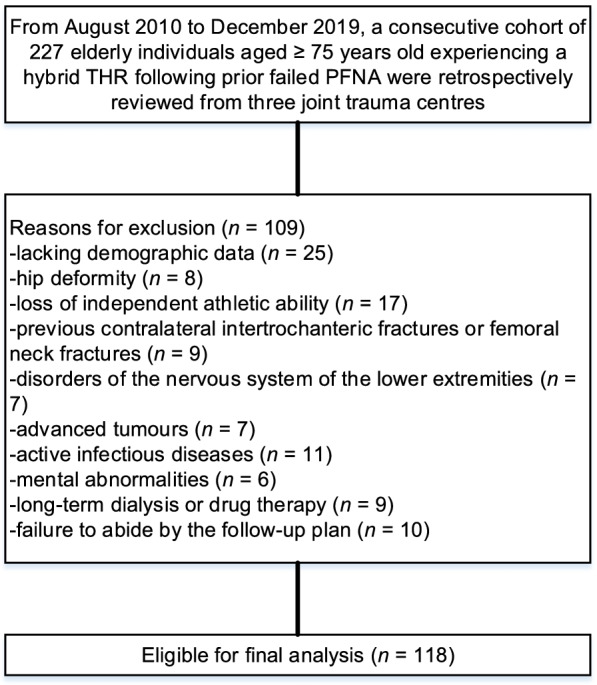
Flow diagram showing the method for the identification of study subjects to estimate the long-term survival of hybrid THRs following prior PFNA failure

In total, 227 elderly individuals aged ≥ 75 years old were included in this study. Among them, 109 individuals were identified as unavailable according to our inclusion criteria, and 118 individuals (118 THRs) were assessed as available, as detailed in Fig. 1. Table 2 shows the baseline data. The age of the patients was primarily concentrated in the range of 75–80 years for 76.3%. In this cohort, there were no noteworthy distinctions in the sex of the patients. The most common mechanism of injury was falling (61.0%), followed by tamp (25.4%) and traffic (13.6%). Most patients had a medium CCI at the time of prosthesis revision, accounting for 58.5%. Indications for conversion to hybrid THR were primarily attributed to instability, accounting for 63.6%. The majority of individuals (57.6%) had an ASA status of 2. The median mHHS prior to conversion was 55.0 (46.7–68.9).

**Table 2:** Patient characteristics at baseline

Variable	Hybrid THR (*n* = 118)
Age (years), no.%	
75 ≤ , < 80	90(76.3)
80 ≤	28(23.7)
Sex, no. %	
Female	60(50.8)
Male	58(49.2)
BMI (kg/m^2^)	
Median (range)	21.9 (18.3–33.6)
BMD (proximal femur) (g/cm^3^)	
Median (range)	3.8(2.9–4.6)
Side, no.%	
Left	55(46.6)
Right	63(53.4)
Reason of primary surgery, no.%	
AO/OTA 31A1.1	27(22.9)
AO/OTA 31A1.2	68(57.6)
AO/OTA 31A1.3	23(19.5)
Mechanism of injury, no.%	
Traffic	16(13.6)
Falling	72(61.0)
Tamp	30(25.4)
Time to THR conversion (months), no.%	
< 6	89(75.4)
≥ 6	29(24.6)
Type of cement fixation, no.%	
Antibiotic-loaded cement	67(56.8)
Cement without antibiotic	51(43.2)
CCI at revision, no. %	
Low	31(26.3)
Medium	69(58.5)
High	18(15.2)
Indications for conversion to hybrid THR, no. %	
Instability	75(63.6)
Mechanical failure	31(26.3)
Both	12(10.1)
ASA physical status, no.%	
1	21(17.8)
2	68(57.6)
3	29(24.6)
mHHS prior to conversion	
Median (range)	55.0(46.7–68.9)

*THR* Total hip replacement, *BMI* Body mass index, *BMD* Bone mineral density, *CCI* Charlson comorbidity index, *ASA* American Society of Anesthesiologists; mHHS: modified Harris Hip Score

### Primary outcome

**Fig. 2:**
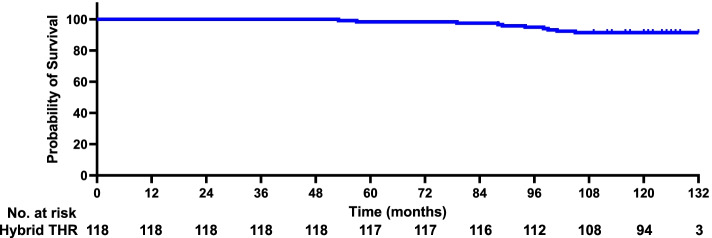
Kaplan–Meier survival curve with revision THR for any reason as the endpoint

The median follow-up was 10 (3–11) years. Figure 2 demonstrates the Kaplan–Meier survival curve with revision THR executed for any reason as the end point. The 5-year survivorship with revision for any reason as the endpoint was 0.983 (95% CI, 0.915–0.992). The 8-year survivorship was 0.949 (95% CI, 0.882–0.975). The 10-year survivorship was 0.914 (95% CI, 0.843–0.960). Of 118 hybrid THRs, 98 (83.1%) were functioning at the end of the study. The most common indication for revision was aseptic loosening (70.0%), followed by periprosthetic fracture (30.0%). In this study, dislocations, even frequent dislocations, did not involve revision, which did not affect the prosthesis survival rate.

### Secondary outcomes

**Fig. 3:**
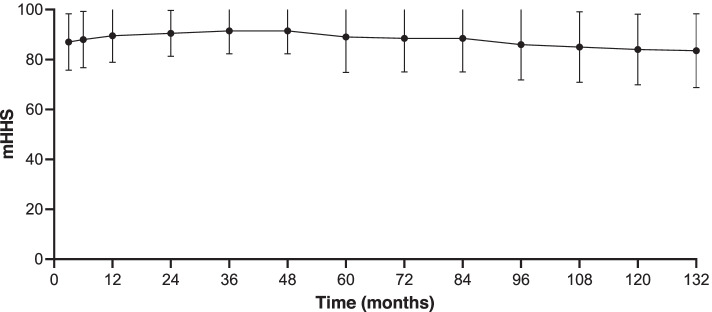
The variation trend of mHHS after conversion to hybrid THRs

Figure 3 provides the variation trend of mHHS after conversion to hybrid THRs. In total, 108 individuals underwent functional outcome assessment, and 10 individuals did not experience a final functional outcome assessment because they underwent revision THR surgery. At the final follow-up, the median function score was 83.6 (79.0–94.0). From the first follow-up after the revision until the 4th year, the curve basically showed an upwards trend. From the 5th year to the 7th year, the curve basically exhibited a horizontal trend. From the 7th year until the final follow-up, the curve presented a downwards trend.

**Fig. 4:**
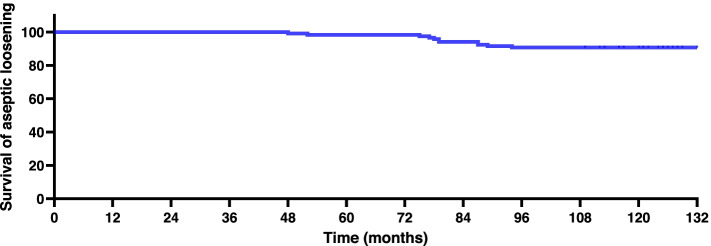
Kaplan–Meier survival curve with aseptic loosening as the endpoint

**Fig. 5:**
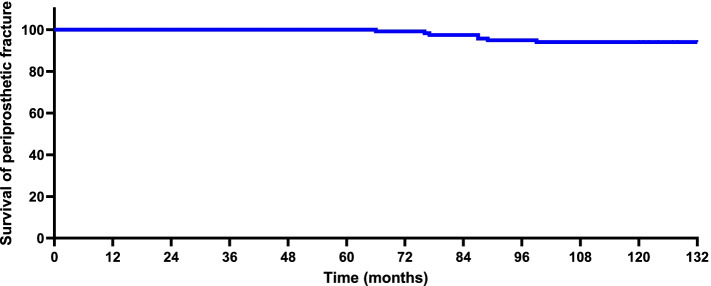
Kaplan–Meier survival curve with periprosthetic fracture as the endpoint

Table 3 shows key implant-related complications. At the final follow-up, 10 (8.4%) individuals underwent a conversion of hybrid THR to revision surgery. The most frequent revision was stem revision (5.1%), followed by acetabular revision (2.5%) and both (0.8%). The most common key complication related to hybrid THR was aseptic loosening (9.3%), followed by dislocation (6.8%) and periprosthetic fracture (5.9%). Figure 4 shows the Kaplan–Meier survival curve with aseptic loosening as the end point, and Fig. 5 shows the Kaplan–Meier survival curve with periprosthetic fractures as the end point. Among the 118 patients included in this study, 16 experienced 26 implant-related complications. The overall incidence of key orthopaedic complications was 13.5% (16/118).

**Table 3:** Key complications related to hybrid THR

Variable, no.%	Hybrid THR (*n* = 118)
Revision (acetabular/stem/both)	3(2.5)/6(5.1)/1(0.8)
Aseptic loosening (stem loosening)	11(9.3)
Dislocation	8(6.8)
Periprosthetic fracture	7(5.9)

*THR* Total hip replacement

## Discussion

Among individuals aged ≥ 75 years old with prior failed PFNA, we found promising long-term results using hybrid THR in revision PFNA surgery. The rate of 10-year survival (based on the need for revision) was 91.4%, which was comparable to previous studies [21, 22]. Our findings may extend those of a limited body of previous studies that have shown a growing rate of 10-year survival in the application of hybrid THR [23]. This increase may be driven by a combination of factors, comprising improvements in bone cement technology and an understanding of the indications for cementing arthroplasty [6, 11]. Hybrid THR may result in a low incidence of studied hip-related complications and substantially good functional outcomes.

Consistent with recent reports [24–26], we did not detect marked distinctions in the 5-year survival rate. The lack of distinctions was most likely attributable to similar follow-up times. However, at the 10-year follow-up, the survival rate was slightly lower than that in previous reports [7, 27–29]. This may be attributed to the fact that most of our subjects had high CCI scores and were afflicted with bone and soft-tissue abnormalities attributed to prior failed PFNA surgery. A high CCI score may reflect a combination of factors that may increase competing risk, such as death [3, 6]. Furthermore, poor survival of hybrid THR was related in part to differences in prosthetic design and bone cement technology used [1, 5, 6, 11]. Although our follow-up span is long, the present study may, to some extent, confirm the advantages of hybrid THR. To date, few studies on converting failed PFNAs provide effective consensus recommendations for reducing or avoiding mechanical failures [30, 31]. Lack of the design and material characteristics of THR implants can lead to remarkable distinctions in comparisons between studies [32, 33]. The long-term outcomes of hybrid THR for elderly individuals remain controversial [21, 27].

It is possible that the most concerning complication is aseptic loosening in the revision PFNA cohort [2, 3]. It is not clear what triggers the aseptic loosening of cement-fixed femoral components, which may involve enlargement of the medullary cavity, metal fatigue, aseptic inflammation at the cement–bone interface, or changes in compressive stress in the proximal femoral cortex [1, 2, 5, 29]. Studies [5, 7, 11, 27] have shown that the acetabulum components and the femoral components differ greatly in the mechanism by which aseptic loosening occurs. The loosening of acetabulum components fixed with bone cement is mostly related to biological effects [3, 21, 27]. Wear debris related to bone cement and polyethylene activate macrophages, release a large number of cytokines, and ultimately result in cytokine-induced osteolysis, thus affecting the stability of acetabular components [34, 35]. In contrast, the loosening of cement-fixed femoral components is mostly related to mechanical effects [36]. Unevenly filled cement and high porosity may affect the riveting force between bone cement and bone [37, 38]. Currently, numerous studies [7, 13, 15, 23] have made hybrid THR the preferred mode of arthroplasty. The femoral components are immobilised with bone cement, irrespective of the effect of enlargement of the medullary cavity over time, which relieves postoperative thigh pain and effectively combats the early sinking and loosening of prostheses, while the acetabulum is fixed with uncemented prosthesis, which can reduce the rate of postoperative loosening [5, 9, 11, 14, 34].

Not all THR prostheses function properly for a long time, and not all individuals benefit from THR revision [1–3]. Any effort to address the increasing incidence of main orthopaedic complications raises the issues of biological characteristics and limited indications of hybrid THR [9, 11, 14, 15]. Implementing a national survey on indications of hybrid THR can be a relatively tricky issue, although there have been encouraging recommendations recently to advocate that large-scale research should be executed to improve this dilemma [12, 13, 16]. However, it is difficult to determine to what extent the growth in THR applications reflects an increase in THR indications or reflects a clinician's personal preference, and current indications for THR may still depend on the clinician's experience [7, 17, 28].

Several limitations should be acknowledged. First, the retrospective design has inherent limitations, such as susceptibility to selection bias and recall bias, the potential for changes in the definition of symptoms and diseases, and the relatively limited data collected (i.e., prefracture functional status). The deviation related to the retrospective design is large and rough, and often due to incomplete data records, it is impossible to explore and discover some relevant factors in depth, or the data records are not sufficiently accurate, resulting in an increase in the error of the obtained data. The present physical and mental state of study subjects may affect the authenticity and accuracy of past data reports, which may be an important defect of retrospective studies. Second, after 5 years of follow-up, the incidence of key orthopaedic complications increased significantly, which is likely to reflect the measurement bias caused by the stricter definition of the main complication variable, rather than a true increase. However, the incidence of aseptic loosening is likely to increase, which may be the result of an interaction between weight gain attributed to the patient's reduced exercise and the biological properties of bone cement. Third, there was at least one competing risk in this study. The use of the Kaplan–Meier method to estimate the risk of cumulative revision may lead to an overestimation of implant survival. Fourth, the nature of the observations makes it impossible for our study to draw reliable causality. Nonetheless, the current study provides an estimate of the long-term survival and complications of hybrid THR, which may be necessary for surgeons' decision-making.

## Conclusion

Hybrid THR may have a remarkable statistical benefit on long-term prosthesis survival, which may reflect the durability of hybrid THR implants, with appropriate mHHS and a low rate of main implant-related complications in the revision setting. We found that the increase in major orthopaedic complications over time may reflect a superposition of a number of underlying factors, including a deterioration in bone quality and an increasing prevalence of cement-related complications. Our findings have contributed to some extent to resolving the debate about the decision-making process in individuals aged ≥ 75 years.

## Data Availability

The datasets generated during and analyzed during the current study are not publicly available due to the protection of patient privacy but are available from the corresponding author on reasonable request.

## Abbreviations

THR: Total hip replacement
PFNA: Proximal femoral nail anti-rotation
mHHS: Modified Harris Hip Score
CIs: Confidence intervals
CCI: Charlson comorbidity index
